# A case of nodular regenerative hyperplasia in a patient who had been taking didanosine

**DOI:** 10.1186/1758-2652-13-S4-P94

**Published:** 2010-11-08

**Authors:** C Jackson, R Fox, M Priest, D Clutterbuck, E Peters

**Affiliations:** 1 Brownlee Center, Infectious diseases, Glasgow, UK; 2 Gartnevel General Hospital, Gastroenterology, Glasgow, UK; 3 Lauriston Building, GUM, Edinburgh, UK

## Purpose of study

It is widely recognised that liver disease causes significant morbidity and mortality in HIV positive patients. Many patients are co-infected with hepatitis B and C, have increased alcohol intake or are on hepatotoxic medication e.g. nevirapine, which can cause acute hepatitis or liver cirrhosis. Nodular regenerative hyperplasia (NRH) is characterised by the presence of diffuse, small regenerative nodules in the absence of significant cirrhosis. Prior use of didanosine is associated with NRH. It leads to portal hypertension, oesophageal varices and subsequent gastrointestinal haemorrhage.

## Methods

A 53-year-old gentleman presented with a major upper gastrointestinal haematemesis. He was diagnosed with HIV 23 years previously and had been on treatment for the last 13 years, maintaining an undetectable viral load and reasonable CD4 count. He denied significant alcohol and a full liver screen was negative. He was taking zidovudine, didanosine and nevirapine from 1997 to 2005, when he was switched to tenofovir, nevirapine and lamivudine. He continued on this combination until 2009 when his tenofovir and lamivudine were replaced with Truvada.

## Summary of results

Liver function tests at the time of admission revealed AST-15, ALT-20, Bilirubin-5, GGT-78. Endoscopy showed bleeding oesophageal varices, which were treated with variceal band ligation (VBL). Despite this he had two further episodes of bleeding, requiring a transjugular intrahepatic portosystemic shunt (TIPPS). Liver biopsy demonstrated focal nodularity within the parenchyma consistent with NRH but no evidence of fibrosis.

## Conclusions

This gentleman had been on didanosine from 1997 to 2005 and it is recognised that this is a risk factor for NRH. It is hypothesised that didanosine, which is a purine analogue, causes destruction of portal veins. Aminotransferases may be normal or elevated. In patients with a previous history of didanosine use, physicians should be aware of the risk of portal hypertension without cirrhosis, which can lead to catastrophic outcomes.

